# Suture of the right internal jugular vein catheter in a mitral valve replacement: a case report

**DOI:** 10.1186/1752-1947-8-129

**Published:** 2014-04-29

**Authors:** Haiying Kong, Shasha Chen, Xiaohong Wen

**Affiliations:** 1 Department of Anesthesiology, the First Affiliated Hospital, Zhejiang University School of Medicine, 79 Qingchun Road, Hangzhou 310003, PR China

**Keywords:** Central venous catheterization, Complications

## Abstract

**Introduction:**

Central venous catheterization can be necessary for patients undergoing a cardiac operation. Accidental suturing of the catheter to the heart is a rare complication that is difficult to correct; excessive traction force on the central venous catheter can lead to heart breakage or even death.

**Case presentation:**

We describe the case of a 56-year-old Han Chinese woman who was scheduled to undergo mitral valve replacement. The central venous catheter placed into her right internal jugular vein was accidentally sutured to the left atrial suture line during the operation. The stuck catheter was successfully removed without having to perform a cardiopulmonary bypass.

**Conclusions:**

Attaching a catheter to the heart by cardiac sutures can occur when the tip of the catheter locates directly above the atrial-caval junction. Care should be taken when closing the cephalad end of a left atrial incision in a mitral valve replacement. Although rare, accidental suturing of the central venous catheter must be kept in mind, and an approach should be chosen to remove the catheter that best avoids additional insult to the heart function.

## Introduction

Central venous catheterization can be necessary for patients undergoing a cardiac operation to obtain central venous access for hemodynamic monitoring (such as of central venous pressure) and administration of some drugs and fluids. The complications of this procedure are well described. Suturing of the catheter to the heart is a rare complication that is difficult to correct [1,2]; excessive traction force on the central vein catheter (CVC) can lead to heart breakage or even death [3]. We report a case of a CVC placed into the right internal jugular vein and accidentally sutured to the left atrial suture line during mitral valve replacement (MVR). The catheter was successfully removed using a reinforcement stitch around the left atrial suture line, without requiring a cardiopulmonary bypass.

## Case presentation

A 56-year-old Han Chinese woman (153cm, 39kg) with severe mitral valve stenosis and moderate mitral valve regurgitation was scheduled to undergo an MVR. She had a four-year history of mitral valve disease. She also had progressive dyspnea on exertion and moderate cardiac dysfunction classified as New York Heart Association class II or III. She was receiving vigorous diuretic and digoxin therapy.

An indirect (Seldinger) technique was used to achieve central catheterization through her right internal jugular vein (Arrow® central venous catheter, 7Fr, 20cm, 0.32 inches, two lumen; Arrow International, Asheboro, NC, USA). No arrhythmia was observed on the monitor while the J wire was advanced. After blood aspiration, the catheter was fixed at the 15cm mark. Her central venous pressure ranged between 7 and 13mmHg throughout the operation and she was transferred to our intensive care unit immediately after surgery.

**Figure 1:**
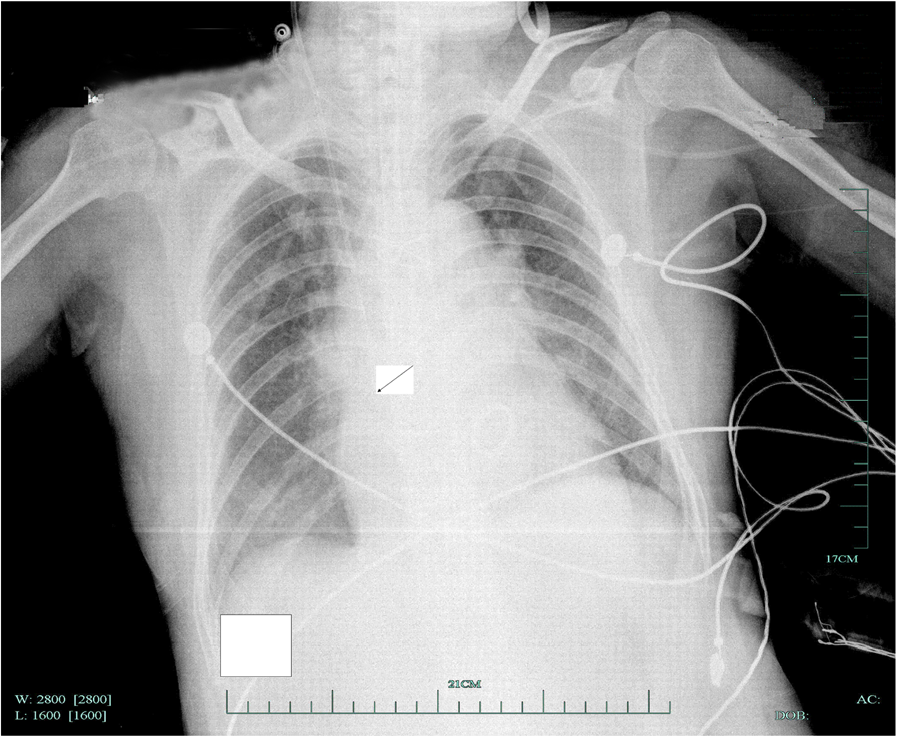
**Anteroposterior chest X-ray.** The right internal jugular cannula is visible. Arrow, catheter tip. Suture of the catheter tip to the heart was highly suspected, and another thoracotomy was scheduled on postoperative day eight. Movement was noted at the interatrial groove incision near the atrial-caval junction while extracting the catheter. Four needles were made with mattress suture around the interatrial groove incision, and the catheter was removed easily after loosening the original threads, which suggested that the catheter had been sutured to the atrial septal tissue. Our patient had an uneventful recovery and the endotracheal tube was extracted the next day.

A control anteroposterior chest radiograph of our patient was obtained on the second day after her operation. This graph revealed that the catheter tip was fixed at the superior vena cava (SVC)-right atrial (RA) junction which caused no attention (Figure 1). Because our patient was unstable, the catheter was not removed and fluid replacement was performed via the CVC. Seven days later, replacement of the catheter was planned for clinically suspected catheter-related sepsis. There was obvious resistance while removing the catheter. Despite gentle traction and clavicular maneuvering, the catheter could not be removed. Additional posteroanterior and lateral chest radiographs were obtained, which showed that the catheter tip was still located in its previous position, the SVC-RA junction.

## Discussion

Accidental suturing of the CVC to the heart is a rare complication. Generally, positioning the tip of the CVC in the atrium, especially near the wall of line, can increase the likelihood of suturing the CVC to the heart. In non-cardiac cases, the tip of the CVC should be located directly above the region where the SVC and the right atrium merge or in the atrium to get accurate central venous pressure values [4,5]. However, the proper position of the CVC tip has not been confirmed in heart surgery, especially in MVR. In our case, the entrapment of the CVC was noted during the MVR surgery, with the CVC tip located directly above the atrial-caval junction. This could partly be explained by our patient’s small atrium and the use of a vertical left atriotomy. During her MVR surgery, an incision was made in her left atrium, posterior to the interatrial groove and anterior to the right pulmonary veins. The incision was extended cephalad and caudal beneath her superior and inferior venae cavae during the difficult exposure of her small left atrium.

In this situation, excessive traction force on the CVC can lead to breakage, although the catheter material can sometimes be faulty and rupture or dilate [3]. Breakage is a lethal complication with an associated mortality of nearly 90% [6,7]. Once entrapment of the CVC is suspected, we advocate very cautious handling of the CVC to avoid this lethal complication.

Various methods such as chest X-ray and transesophageal echocardiography can be used to confirm the catheter tip position [8]. Transesophageal echocardiography has been shown to accurately monitor the position of the catheter tip at the SVC-RA junction [9], and it provides a more sensitive assessment than chest radiography, which does not reliably identify the position of catheter tips at the SVC-RA junction. However, transesophageal echocardiography requires special equipment that was not available in our hospital. Although repeated chest radiograph imaging showed that the CVC tip was located at the SVC-RA junction, suture of the CVC tip to her heart was still suspected after the difficult removal of the CVC.

Patients who receive MVR generally have poor heart function before surgery, and inevitably experience ischemia-reperfusion injury during cardiopulmonary bypass. They have low tolerance to additional insults to heart function, which can have a detrimental impact on short-term and long-term outcomes. Hence, the surgical approach to remove the CVC should be carefully chosen. In our case, a reinforcement stitch was placed around our patient’s left atrial suture line and the CVC was delivered into her atrium after loosening the original suture lines.

## Conclusion

Attaching the CVC to the heart by cardiac sutures can occur accidentally when the tip of the CVC locates directly above the atrial-caval junction. Care should be taken while closing the cephalad end of a left atrial incision in MVR. The SVC and the right atrium should be well retracted. If there is any doubt, the anesthetist should gently pull the CVC before the left atrial suture line is tied. Although rare, suture of the CVC must be kept in mind, and an approach should be chosen to remove the CVC that best avoids additional insult to heart function.

## Consent

Written informed consent was obtained from the patient for publication of this case report and any accompanying images. A copy of the written consent is available for review by the Editor-in-Chief of this journal.

## Abbreviations

CVC: central vein catheter; MVR: mitral valve replacement; RA: right atrial; SVC: superior vena cava.

## Competing interests

The authors declare that they have no competing interests.

## Authors’ contributions

HYK and SSC carried out the anesthesia. HYK wrote the manuscript and XHW helped to draft the manuscript. All authors read and approved the final manuscript.
